# Functional diversity of apolipoprotein E: from subcellular localization to mitochondrial function

**DOI:** 10.1007/s00018-022-04516-7

**Published:** 2022-08-26

**Authors:** Johanna Rueter, Gerald Rimbach, Patricia Huebbe

**Affiliations:** Devision of Food Science, Institute of Human Nutrition and Food Science, University of Kiel, Hermann-Rodewald-Strasse 6, 24118 Kiel, Germany

**Keywords:** Mitochondria-associated ER membranes, Protein–protein interactions, Interactome, Proteolytic fragmentation

## Abstract

Human apolipoprotein E (APOE), originally known for its role in lipid metabolism, is polymorphic with three major allele forms, namely, *APOEε2*, *APOEε3*, and *APOEε4,* leading to three different human APOE isoforms. The *ε4* allele is a genetic risk factor for Alzheimer’s disease (AD); therefore, the vast majority of APOE research focuses on its role in AD pathology. However, there is increasing evidence for other functions of APOE through the involvement in other biological processes such as transcriptional regulation, mitochondrial metabolism, immune response, and responsiveness to dietary factors. Therefore, the aim of this review is to provide an overview of the potential novel functions of APOE and their characterization. The detection of APOE in various cell organelles points to previously unrecognized roles in mitochondria and others, although it is actually considered a secretory protein. Furthermore, numerous interactions of APOE with other proteins have been detected, providing indications for new metabolic pathways involving APOE. The present review summarizes the current evidence on APOE beyond its original role in lipid metabolism, to change the perspective and encourage novel approaches to future research on APOE and its isoform-dependent role in the cellular metabolism.

## Introduction

Apolipoprotein E (APOE) is a plasma protein involved in cholesterol metabolism that, as a component of lipoproteins, mediates their cellular uptake by binding to cell surface receptors. In humans, there are three different isoforms resulting from the three allelic variants *ε2*, *ε3,* and *ε4*. *APOEε4* carriers show an increased risk of the development of Alzheimer’s disease (AD), which is why a major focus of APOE research is on the relationship between APOE and AD pathology, and most studies focus on the role of APOE in the brain or neuronal cells and astrocytes [1–3]. However, APOE is mainly produced in the liver, and to a lesser extent in astrocytes and microglia. There is much evidence that APOE circulates in two independent pools, since APOE is not able to cross the blood–brain barrier [4], APOE protein levels in plasma are significantly higher than in cerebrospinal fluid (CSF) [5], and plasma and CSF APOE have different posttranslational modifications [6]. Therefore, it must be considered that findings on the role of APOE in the brain may not be directly transferable to APOE in the periphery.

While the *ε4* allele shows detrimental effects in the elderly, carrying the *ε4* allele actually is beneficial in some aspects, e.g., improved outcome of certain infectious diseases or increased vitamin D levels [7–9]. Therefore, the question arises as to what functions APOE might have in addition to lipid transport and its role in AD pathology. A number of physiological processes in which APOE is involved have already been described, e.g., participation in the immune system [10, 11] or in the response to various nutritional factors [9, 12, 13] reviewed in Egert et al. [14], as well as its binding to a number of genes and thus its function as a transcription factor [15]. Insights into further novel metabolic participations of APOE can be obtained by studying its physical interactions with other proteins. In addition, previously unknown functions may be deduced from the subcellular localization of APOE, since APOE has been detected in various cell organelles although it is primarily a secretory protein [15–17]. Therefore, this review discusses the following topics: The subcellular localization of APOE and proteolytically cleaved APOE fragments, and their resulting roles in mitochondrial metabolism and mitochondria-associated ER membranes (MAMs). The second focus is the interaction of APOE with other proteins, whose function is well understood, and the identification of signalling pathways in which APOE is involved through these interactions.

## Protein structure, subcellular trafficking, and cellular processing of APOE

### APOE protein structure and isoforms

**Fig. 1:**
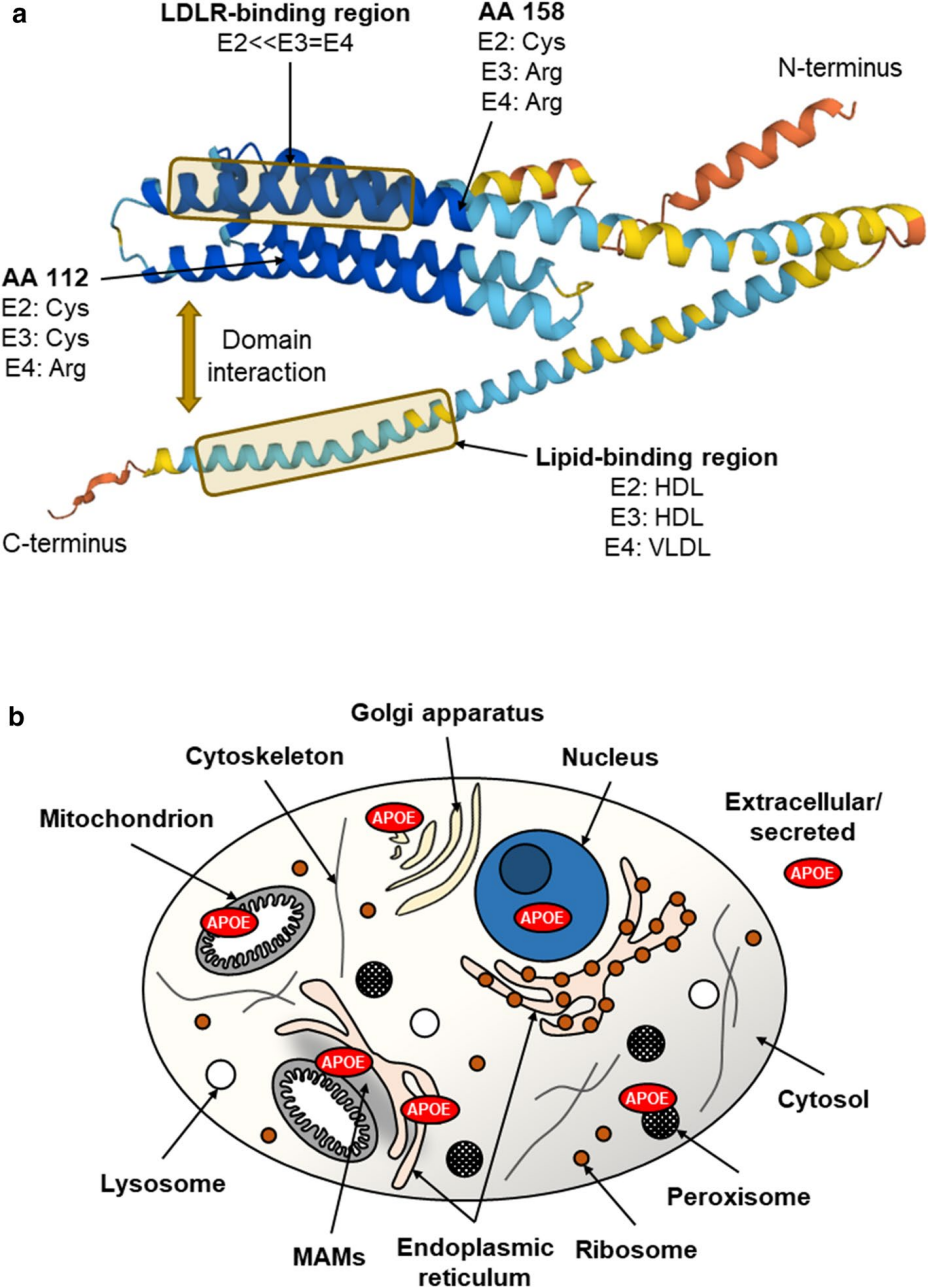
Predicted spatial protein structure of human APOE (AlphaFold) and intracellular localisation of APOE. **a** The APOE isoproteins differ at one and two amino acid (AA) positions, respectively (112 and 158). This affects the structure and thus the function of the protein, e.g., APOE2 shows a significantly reduced affinity for the low-density lipoprotein receptor (LDLR) and the lipid binding preference differs between the isoforms. The different colours of the protein structure indicate the model confidence of the structure prediction (dark blue, very high; light blue, confident; yellow, low; orange, very low). Protein structure image downloaded from https://alphafold.ebi.ac.uk/. *HDL* high-density lipoprotein, *VLDL* very low-density lipoprotein. **b** Schematic illustration of an eukaryotic cell and the individual cell organelles and compartments. APOE is known to be a secreted protein, but has been detected inside the cell in, e.g., mitochondria, peroxisomes, and the nucleus. *MAMs* mitochondria-associated ER membranes

Expression of the human *APOE* gene results in a 317-amino-acid-long APOE precursor protein, from which the mature APOE protein with a length of 299 amino acids is formed after cleavage of an N-terminal 18-amino-acid-long signal peptide [18]. The nucleotide exchanges (rs429358 and rs7412) leading to the three main human APOE isoforms result in amino acid exchanges at positions 112 and 158, which are located in the N-terminal domain of the mature protein (Fig. 1a). The APOE4 protein carries arginine residues at both positions, APOE3 has cysteine at position 112 and arginine at position 158, and APOE2 carries cysteine at each of the corresponding positions [19]. These polymorphism of APOE is found exclusively in humans, although APOE is expressed in mammals and other vertebrates [20, 21]. There are some differences in the structural properties of the APOE isoforms that are likely due to the exchange of amino acids, e.g., the domain interaction between the N- and C-termini appears to be affected by the exchange at position 112, such that APOE4 exhibits greater dynamics in structure opening [22, 23]. Furthermore, the stability to chemical denaturation (stability E4 < E3 < E2) and self-association differ between the isoforms [24–26]. These structural differences could have consequences for possible further functions of APOE, since due to the different spatial structures of the isoforms, different regions of the amino acid sequence might be exposed or buried, which could influence, e.g., interactions with other molecules.

Because *APOEε2* is the least common gene variant, there are only few studies examining this genotype, especially in the context of mitochondrial function which is why the focus of this article is on APOE3 and APOE4.

### Subcellular localization of APOE

The APOE protein is a secretory protein but has also been localized inside the cell in many subcellular organelles and compartments (Fig. 1b), such as mitochondria, peroxisomes, and the nucleus [15–17]. Secretory proteins such as APOE contain an amino acid sequence that is recognized by the signal recognition particle and thereby translated directly into the ER. Intracellular proteins are translated from cytosolic ribosomes into the cytosol and contain peptide sequences that are coexpressed and linked to the actual protein sequence at the C- or N-terminus. Examples are the nuclear localization sequence (NLS) or the mitochondrial localization sequence (MLS), which are recognized by proteins on the corresponding membranes that mediate transport into the nucleus or mitochondrion. The APOE protein has been shown to be present in the nucleus and to function as a transcription factor [15, 27], but no NLS has yet been identified in the APOE protein sequence that could explain the nuclear import mechanism of APOE. Similarly, there is much evidence that APOE is present in mitochondria and affects mitochondrial function [16, 17, 28, 29]. However, no MLS has yet been identified in the protein sequence of APOE; consequently, there must be other mechanisms or pathways by which APOE enters the nucleus and passes through mitochondrial membranes into mitochondria. In addition to the localization of APOE in mitochondria, APOE was also detected in MAMs [17, 30]. An overview of the evidence of localization of APOE in these two cell organelles is provided in Table 1. One pathway by which APOE might enter the mitochondria is through interorganelle crosstalk between the endoplasmic reticulum (ER) and mitochondria, as has recently been shown for glycoproteins and misfolded proteins [31]. The secretion of misfolded and structurally altered ER-cargo into mitochondria, may explain the occurrence of APOE fragments in the mitochondrion [32]. Proteolytic cleavage of APOE yields fragments with altered folding characteristics, which can be recognized as misfolded and thus transferred to mitochondria for sequestration. However, this is highly speculative and must be investigated in the future.

**Table 1:** Experimental evidence for the localization of APOE in mitochondria and mitochondria-associated ER membranes (MAMs)

Cellular component	Tissue/cell type	Isolation/labelling	Detection	References
**Mitochondria**	Rat liver	Immunogold labelling of APOE in cryosections	Electron microscopy	[16]
	Rat liver	Radioactive leucine labelling and Percoll density gradient ultracentrifugation	Anti-VLDL immunoprecipitation, SDS gel electrophoresis, excision of protein bands and radioactivity counting	[17]
	APOE4-transfected Neuro2a cells	Not described^1^	Western blotting of mitochondrial fractions	[33]
	APOE TR mouse hippocampus	Isolation using a commercially available kit ^2^ and differential centrifugation	Western blotting, LC–MS	[28]
	APOE TR mouse primary astrocytes	Coimmunostaining of Mito-GFP and APOE Isolation using a commercially available kit ^3^ and differential centrifugation	Fluorescence microscopy Western blotting of mitochondrial fractions	[29]
**Mitochondria-associated ER membranes**	Rat liver	Radioactive leucine labelling and Percoll density gradient ultracentrifugation	Anti-VLDL immunoprecipitation, SDS gel electrophoresis, excision of protein bands and radioactivity counting	[17]
	Mouse liver and brain	Percoll density gradient ultracentrifugation	LC–MS	[34]
	Huh7 cells	Percoll density gradient ultracentrifugation	LC–MS	[35]
	Mouse liver	Percoll density gradient ultracentrifugation	LC–MS	[30]
	Mouse brain	Percoll density gradient ultracentrifugation	LC–MS	[36]
	Mouse liver	Sucrose gradient centrifugation^4^	LC–MS	[37]

APOE has been repeatedly detected in mitochondria and MAMs in rat, mouse, and human cultured cells and tissue using various methodological approaches

*TR* targeted replacement, *LC–MS* liquid chromatography–mass spectrometry

^1^“Mitochondrion-rich fractions” were isolated, but the isolation method was not described

^2^Benchtop mitochondria isolation kit for rodent tissue (Mitosciences)

^3^ProteoExtract cytosol/mitochondria fractionation kit (EMD Millipore)

^4^Analysis of rough ER wrapped around mitochondria, which include MAMs as a subdomain

### Proteolytic fragmentation of APOE

APOE fragments are the result of proteolytic cleavage, which is a normal cellular process to degrade proteins that are abnormal or no longer needed and to regulate cellular processes, e.g., activation and inactivation of enzymes by proteolysis. In this process, smaller fragments are formed from a protein, and when fully degraded, the individual amino acids can be recycled. For APOE, fragments of varying length (a few amino acids up to 29 kDa) have been described to be formed by proteolytic cleavage of various proteases, e.g., high-temperature-requirement serine peptidase A1 [38]. Overall, many of the identified APOE fragments are C-terminally truncated fragments found in the brain, where they tend to have detrimental effects and are often associated with AD. Munoz et al. [38] provided a detailed overview of the APOE fragments in different cells and animal models related to AD [38]. These fragments have different physiological functions, some of which have cytotoxic effects [39], decrease Aβ clearance [40], or accumulate in AD brains and cause AD-like neurodegeneration in mice [41].

Independent of AD, there are some fragments related to mitochondria: a 29-kDa fragment (amino acids 1–272) showed increased ER stress, altered composition of MAMs, impaired mitochondrial function, and interaction with mitochondrial proteins [32, 33, 42]. According to Chang et al. [32], APOE4 fragments have a neurotoxic effect in Neuro2a cells, which the authors suggest is due to a mislocalization in mitochondria and associated mitochondrial dysfunction. No conclusions can be drawn about APOE3 fragments, as these have not been studied [32]. A more specific interaction of APOE fragments with mitochondria was demonstrated in Neuro2a cells, where it was shown that a 29-kDa APOE4 fragment (amino acids 1–272) binds directly to mitochondrial respiratory chain proteins and decreases the activity of complexes III and IV to a greater extent than full-length APOE4. APOE3 fragments were not analysed [33]. The 29-kDa fragment lacks a part of the C-terminus involved in domain interaction (amino acids 270–290), which probably results in the spatial structure of this fragment being quite different compared to that of the full-length APOE protein. The altered structure may, for example, expose regions of the amino acid sequence that are normally hidden in full-length APOE, potentially creating binding sites in the APOE fragments for interactions with other molecules that are not present in full-length APOE. In addition to the mitochondrial interaction and localization of APOE fragments, APOE fragments were also shown to be localized in the nucleus, indicating potential activity as a transcription factor, as observed with a 17-kDa APOE fragment [43, 44]. Most studies on APOE fragments conclude that the detected APOE fragments have adverse effects, except for a study in which an extracellular 25-kDa APOE fragment was found to act neurotrophically and to stimulate neurite growth in cultured neurons [38]. Little is known about APOE fragments in other tissues and nonneuronal cells. There is one study on the presence of a 22-kDa APOE fragment in cultured hepatocytes that binds to secreted very low-density lipoprotein (VLDL) [45]. Another category of fragments are small peptides that are only 17–30-amino-acids long and have antimicrobial activity. These peptide sequences originate from the middle of the amino acid sequence of the APOE protein, e.g., they are found in the low-density lipoprotein receptor (LDLR) or heparin binding region [46, 47].

Since an association between APOE fragments and AD is often postulated, one might assume that APOE fragments in CSF could be used as biomarkers for AD. However, no correlation was found between AD or *APOE* genotype and the concentration of an APOE fragment in CSF or plasma [48]. Overall, the question is whether these APOE fragments, most of which have shown adverse effects, have a function in the brain or whether they are waste products that have not been properly broken down and sometimes happen to have toxic effects. Furthermore, it is unclear whether the same fragments found in the brain are also found in other tissues, such as the liver, and whether they show similar or different effects there.

Based on the growing evidence for the presence of full-length APOE and APOE fragments in mitochondria and MAMs, the potential function of APOE in these two cell organelles will be further addressed in the next two sections.

## APOE and its potential role in the mitochondrion

### Mitochondrial localization of APOE

The first evidence of mitochondrial APOE dates back to the early 1990s. Immunogold labelling of APOE in rat hepatocytes identified APOE in peroxisomes and the Golgi apparatus but also small amounts of APOE in mitochondria [16]. At the same time, Vance [17] showed that apolipoproteins were enriched in mitochondria, MAMs, and microsome fractions isolated from rat liver, with APOE being the most abundant apolipoprotein. Further evidence for the mitochondrial localization of APOE is provided by an analysis of the mitochondrial hippocampus proteome of APOE3 and APOE4 targeted replacement (TR) mice. Remarkably, in APOE4 mice, APOE protein levels were significantly increased in mitochondria after ischaemic damage, which may indicate stress-dependent mitochondrial translocation [28]. Then, APOE was again shown to accumulate in the mitochondrial fraction of primary astrocytes of APOE TR mice, and colocalization was detected by immunostaining of APOE and mitochondria by confocal microscopy [29]. However, the mechanism or pathway by which APOE enters the mitochondrion remains to be elucidated. The import of molecules into the mitochondrion depends on their size. While small molecules such as calcium and ATP can cross the outer mitochondrial membrane (OMM) through voltage-dependent anion-selective channel 1 (VDAC1), larger molecules such as proteins require the abovementioned MLS to enter the mitochondrial membrane via the translocase of the outer mitochondrial membrane (TOM) complex [49]. In addition to importing small molecules, VDAC1 also plays a role in the protein import of the TOM complex by acting as a coupling factor between the TOM and translocase of the inner mitochondrial membrane (TIM) complex subunits in the mitochondrial intermembrane space [50]. Interestingly, APOE was shown to interact with VDAC1 [51, 52]; however, APOE would still be too large to be imported through VDAC1 channels.

### Effects of APOE on mitochondrial function and dynamics

The main function of mitochondria is energy production in the form of ATP via the tricarboxylic acid (TCA) cycle in the mitochondrial matrix and oxidative phosphorylation in the inner mitochondrial membrane (IMM). Altered ATP levels depending on *APOE* genotype could indicate an influence of APOE on mitochondrial function. This notion was investigated in several studies in APOE-transfected Huh7 cells [53], primary astrocytes isolated from APOE TR mice [29], primary neurons isolated from APOE TR mice [54], and in the brains of APOE TR mice [12], with the result of higher ATP levels in APOE3 cells and mice compared to APOE4. Yin et al. [55] suggested that APOE affects mitochondrial function via the peroxisome proliferator-activated receptor gamma coactivator 1-α (PGC1α) and sirtuin 3 (SIRT3) pathways, as APOE4 mice exhibited reduced PGC1α and SIRT3 expression [55]. However, there are also studies that found no difference between APOE isoforms in total ATP levels but differences in the source of ATP, e.g., higher ATP levels generated by oxidative phosphorylation (OXPHOS) and lower glycolysis-derived ATP in primary endothelial cells from APOE4 mice [56]. Others distinguished between basal and stressed states and found significantly lower ATP levels in stressed APOE4 cells than in APOE3 cells [57].

One reason for the altered ATP levels could be a disruption in the respiratory chain or in the individual complexes of the electron transport chain; therefore, the effect of the *APOE* genotype on the protein levels of OXPHOS complexes has been studied intensively [53, 57, 58]. The protein levels of complexes I–V were reduced in APOE4 neurons compared to APOE3 neurons [54, 57, 58], while there was no significant difference in astrocytes [57]. In contrast to the decreased protein levels in APOE4 neurons, some subunits of complexes I–V in the entorhinal cortex of APOE3/4 TR mice were increased at the mRNA level compared to APOE3/3 [59]. There are only a few studies examining the effect of APOE on mitochondrial respiration outside the brain, but these studies tend to show no difference between APOE3 and APOE4. In the liver of APOE TR mice and in APOE-transfected Huh7 cells, no isoform-dependent effect on OXPHOS protein levels was found [53], and rat cardiomyocytes treated with recombinant APOE3 and APOE4 showed almost no difference in mitochondrial respiration [52]. The effect of APOE on OXPHOS appears to be dependent not only on the tissue studied (brain versus periphery) but also on the different cell types within the brain. For example, differences in mitochondrial respiration depending on the APOE isoform were observed in the hippocampus but not in the entorhinal cortex [59], and the results are also partially contrasting in neuronal cells and astrocytes [54, 58].

Mitochondria undergo structural changes by fission and fusion and are, therefore, highly dynamic cell organelles. In mitochondrial fusion, two or more organelles become one larger one, induced by specific proteins such as mitofusin 1 and 2 (MFN1/2) and dynamin-like 120 kDa protein (OPA1). In fission events, one mitochondrion is split into two, mediated by proteins including dynamin-related protein 1 (DRP1) and mitochondrial fission 1 protein (FIS1) [60]. APOE appears to have an effect on mitochondrial fusion and fission, although studies show partially conflicting results. In the hippocampus and primary astrocytes of APOE TR mice and human postmortem brain tissue of AD patients, increased mitochondrial fusion was observed with APOE4 [29, 57, 61]. In contrast, in human postmortem brain tissue, both fusion and fission proteins were downregulated in *APOEε4* carriers compared to non-*APOEε4* carriers (independent of AD status), indicating overall reduced mitochondrial dynamics [62]. APOE-associated with a specific form of low-density lipoprotein (L5-LDL) influences the phosphorylation of DRP1, which leads to an increase in mitochondrial fission in rat heart cells (H9c2 cells), independent of the APOE isoform [52]. An examination of markers of mitochondrial dynamics in Huh7 cells and liver of APOE TR mice also revealed no isoform-dependent differences at the mRNA level (MFN2 and FIS1) [53]. In addition to the possible effects of the APOE isoform, APOE fragments also appear to have an impact on mitochondrial dynamics. Compared with full-length APOE4, a 29-kDa APOE4 fragment showed increased mitochondrial fragmentation and greater fission in the hippocampus of APOE TR mice and neuronal cells. Conclusions on isoform-dependent differences could not be made because APOE3 fragments were not examined [42]. Further evidence of the possible involvement of APOE in mitochondrial metabolism is provided by experiments with APOE knockout mice. Analysis of the liver and hippocampal mitochondrial proteome of APOE knockout mice showed a significant change in the expression of some mitochondrial proteins, such as the subunits of ATP synthase and hexokinase 1 [63, 64].

In summary, there are many indications from different methodological approaches that APOE exhibits a prominent function in the mitochondria (Table 2). However, depending on the tissue and cell type, the APOE isoforms seemingly exhibit different effects on the respiratory chain, ATP levels, and mitochondrial dynamics, which warrants further investigation. This is of particular importance, since APOE is not able to cross the blood–brain and thus, two different APOE pools exist in the human body (brain and periphery), in which the APOE protein shows different concentrations and posttranslational modifications [4–6]. In addition, there is much evidence of proteolytically cleaved APOE fragments in the brain, but almost no studies on fragments in other tissues [38]. This could also lead to tissue-specific APOE isoform-dependent differences. It should be considered that most of the studies on APOE and its mitochondrial involvement were performed in the brain or neuronal cells. However, these results may be not directly transferable to peripheral APOE.

**Table 2:** Overview of the APOE isoform-dependent effects on mitochondrial and MAM function with the tissue or cell line studied (brain or periphery) indicated

	Readout		Isoform effect	Tissue source	References
**Mitochondria**	Total ATP level		E3 > E4	Brain (APOE TR mice)	[12]
				Periphery (Huh7 cells^1^)	[53]
				Brain (temporal lobes of APOE TR mice)	[55]
				Brain (primary astrocytes of APOE TR mice)	[29]
				Brain (primary neurons of APOE TR mice)	[54]
			E3 < E4	Brain (entorhinal cortex of APOE TR mice)	[59]
				Brain (primary astrocytes of APOE TR mice)	[54]
			Basal: E3 = E4 Stress: E3 > E4	Brain (Neuro2a cells^2^)	[57]
			E3 = E4	Brain (primary endothelial cells of APOE TR mice)	[56]
	OXPHOS	Protein	E3 > E4	Brain (primary neurons of NSE-APOE mice)	[58]
				Brain (cortical synaptosome of APOE TR mice)	[65]
				Brain (Neuro2a cells^2^)	[57]
				Brain (primary neurons of APOE TR mice)	[54]
			E3 = E4	Brain (primary astrocytes of GFAP–APOE mice)	[58]
				Periphery (Huh7 cells^1^, liver from APOE TR mice)	[53]
			E3 < E4	Brain (primary astrocytes of APOE TR mice)	[54]
		mRNA	E3/3 < E3/4	Brain (entorhinal cortex of APOE TR mice)	[59]
	PGC1α pathway	Protein	E3 > E4	Brain (temporal lobes of APOE TR mice	[55]
				Brain (primary neurons of APOE TR mice)	[54]
			non-E4 > E4	Brain (human postmortem brain tissue)	[62]
		mRNA	E3 > E4	Brain (cortex from APOE TR mice)	[12]
			E3 = E4	Periphery (Huh7 cells^1^, liver from APOE TR mice)	[53]
	Mitochondrial respiration (Seahorse)		E3 > E4	Brain (Neuro2a cells^2^)	[58]
				Brain (primary neurons of APOE TR mice)	[54]
			E3 = E4	Periphery (H2c9 cells, treated with recombinant APOE)	[52]
			Depends on age, brain area and complex	Brain (entorhinal cortex and hippocampus of APOE TR mice)	[59]
	Mitochondrial membrane potential		E3 = E4	Periphery (Huh7 cells^1^)	[53]
			E3 > E4	Brain (primary astrocytes of APOE TR mice)	[29]
				Brain (primary neurons of APOE TR mice)	[54]
	Mitochondrial translocation/accumulation		E3 < E4 (stress-induced)	Brain (hippocampus of APOE TR mice)	[28]
			E3 < E4	Brain (primary astrocytes of APOE TR mice)	[29]
**Mitochondria-associated ER membranes**	MFN1/2	Protein	E3 < E4	Brain (Neuro2a cells^2^)	[57]
				Brain (hippocampus of APOE TR mice)	[61]
				Brain (primary astrocytes of APOE TR mice)	[29]
			non-E4 > E4	Brain (human postmortem brain tissue)	[62]
		mRNA	E3 = E4	Periphery (Huh7 cells^1^, liver from APOE TR mice)	[53]
	GRP75	Protein	GRP75c: E3 < E4 GRP75d: E3 > E4	Brain (hippocampus from APOE TR mice)	[66]
			E3 = E4	Brain (Neuro2a cells^2^)	[58]
			E3 > E4	Brain (Neuro2a cells^2^)	[57]
	VDAC1	Protein	E3 = E4	Brain (Neuro2a cells^2^)	[58]
			E3 < E4	Brain (Neuro2a cells^2^)	[57]
		mRNA	E3 < E4	Brain (Neuro2a cells^2^)	[58]
			E3 = E4	Periphery (Huh7 cells^1^, liver from APOE TR mice)	[53]
	Phospholipid synthesis		E3 < E4	Brain (mouse hippocampus cells treated with APOE-containing astrocyte-conditioned media)	[67]
	ER-mitochondria calcium flux		E3 < E4	Brain (Neuro2a cells^2^)	[57]
	ER-mitochondria colocalization		E3 < E4 (in trend)	Brain (mouse hippocampus cells treated with APOE-containing astrocyte-conditioned media)	[67]
	VLDL assembly		-	Periphery (liver from CAV1 knockout mice)	[30]
				Periphery (mouse liver)	[37]

^1^Transient APOE3- and APOE4-transfected cells

^2^Stable APOE3- and APOE4-expressing cell lines

## Role of APOE in mitochondria-associated ER membranes

**Fig. 2:**
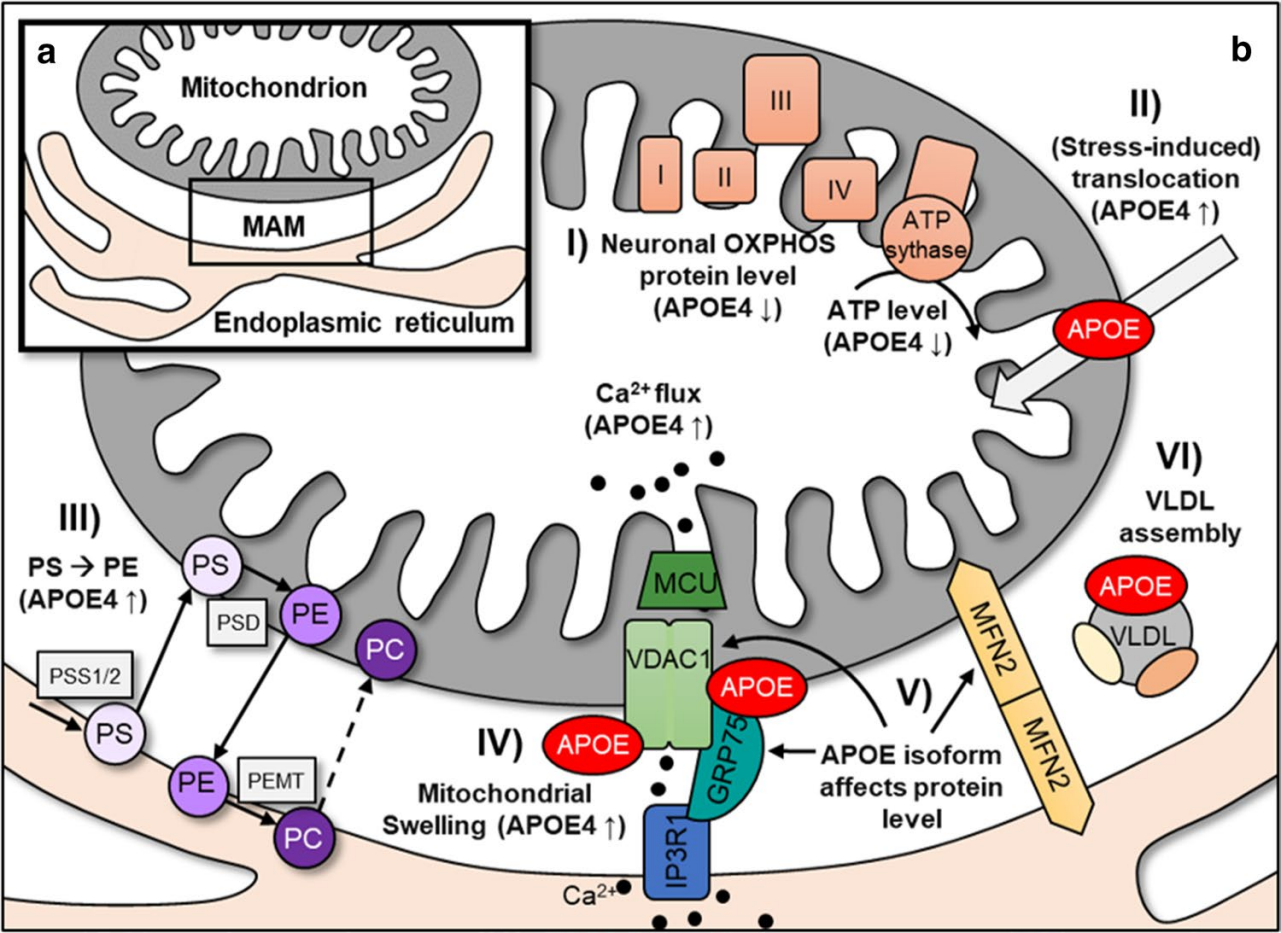
Illustration of the assembly of mitochondria and mitochondria–ER contacts and the inherent biochemical pathways with suggested APOE involvement or regulation by the APOE isoform. **a** Mitochondria-associated ER membrane complex (MAM). **b** Mitochondrial and MAM pathways in which APOE may be involved. (I) The impact of APOE isoforms on mitochondrial function depends on the cell type and species, but consistently decreased neuronal OXPHOS protein and ATP levels were observed in APOE4. (II) Mitochondrial accumulation and stress-induced translocation are increased in APOE4. (III) The different steps in phospholipid synthesis take place through consecutive exchange of substrates from the ER membrane (synthesis of phosphatidylserine; PS) to the mitochondrion (conversion of PS to phosphatidylethanolamine (PE), which is increased in APOE4) and back. The final methylation step by PEMT is accomplished in the ER membrane, yielding phosphatidylcholine (PC). (IV) Calcium is released from the ER through the IP3R1–GRP75–VDAC1 complex and shuttled to the mitochondrion through the mitochondrial calcium uniporter protein (MCU) into the inner matrix. Increased calcium flux was found in APOE4 Neuro2a cells, and higher mitochondrial swelling was observed in APOE4-treated H9c2 cells, which was caused by the interaction of APOE (derived from the lysosomal degradation of L5-LDL) with VDAC1. The protein–protein interaction of APOE with MAM proteins such as VDAC1 and GRP75 is one explanation for the presence of APOE in MAMs. (V) MFN2 dimers connect the OMM with the ER membrane, acting as MAM tethering proteins. Depending on the tissue and species, APOE affects the expression of MFN1 and MFN2. (VI) MAMs are involved in cholesterol metabolism, and proteomic analyses provide evidence for a possible role of APOE in VLDL assembly in MAMs

Mitochondria interact not only with other mitochondria but also with other organelles, such as the ER. These interactions of the OMM and the ER membrane are described as MAMs and/or mitochondria–ER contacts, so-called “MERCs” (Fig. 2a). These definitions are sometimes used synonymously, although distinctions must be made, since MAMs are the result of subcellular isolation and fractionation of membranes that are associated with mitochondria and ER, and MERCs are the structural and functional organization of interorganelle contacts [68]. For a better understanding, only the term "MAMs" is used in this article.

### Proteome of mitochondria-associated ER membranes and function of mitochondria–ER contacts

The contacts between mitochondria and the ER have been studied for many decades. In 1990, Vance [17] isolated a membrane fraction associated with mitochondria that had many similarities to microsomes but showed a unique and significantly different protein profile compared to mitochondria and ER. This fraction was declared as “fraction X” and later as MAMs [17, 69]. The mammalian MAM protein composition was analysed by mass spectrometry (MS) for the first time by Zhang et al. [70], with an increasing number of proteomic studies analysing MAM fractions in different tissues and cells that have followed [30, 34–36]. From numerous studies in which the MAM proteome has been examined, it has become clear that the MAMs are a dynamic cell structure that is influenced by various parameters, including ageing, diseases such as diabetes, and nutritional factors [36, 71–73]. In addition, the MAM proteome differs by tissue type (liver and brain) [34].

The MAM resident proteins can be classified according to their function. There are tethering proteins that maintain the connection and the distance between the ER and OMM, such as MFN1 and MFN2 [74], which are also important in mitochondrial fusion. Apart from these classical MAM proteins, the assembly of MAMs is very flexible, and proteins can be recruited depending on the state of the cell [75]. Further evidence for the flexibility of MAM assembly is provided by an experimental approach using a synthetic GFP-tagged MAM linker, which demonstrated that contacts between mitochondria and ER can change within minutes and that various stressors, such as starvation and apoptosis, increase the number of contacts [76].

Mitochondria–ER contacts are important in all processes where molecules need to be shuttled between the ER and mitochondria, such as during phospholipid synthesis, since it takes place in both, the OMM and the ER membrane (Fig. 2b) [69, 77]. In addition to lipids, calcium is also transported between the ER (most important cellular calcium storage site) and mitochondria. Calcium is released from the ER through the inositol 1,4,5-trisphosphate receptor 1 (IP3R1) ion channel, crosses the OMM through VDAC1 and accumulates in the mitochondrial matrix by passing through the IMM by way of the mitochondrial calcium uniporter protein (MCU) [78]. The chaperone protein glucose-regulated protein 75 (GRP75) forms a bridge between IP3R1 and VDAC1 [79, 80] and thus, the IP3R1–GRP75–VDAC1 complex has a crucial role in maintaining calcium homeostasis in mitochondria (Fig. 2b).

Glucose appears to influence mitochondria–ER contacts, as high glucose (25 mM) treatment reduced VDAC1–IP3R1 contacts in cultured hepatocytes by half compared to those of low glucose (5 mM) treatment. Similar effects have been observed in hepatocytes from *ob/ob* mice (leptin-deficient mice, exhibit obesity and glucose intolerance) [71, 72] and myotubes of obese and diabetic humans, wild-type mice and healthy subjects, respectively [81]. Overall, the protein composition of MAMs and the integrity of mitochondria–ER contacts seem to be important for normal insulin signalling [36, 71]. MAMs may likewise play a significant role in neuronal disease pathology such as AD, since it has been observed that the level of presenilins and γ-secretase activity was increased in MAMs of AD patients, while phospholipid synthesis in fibroblasts was also higher [82, 83]. Interestingly, amyloid beta (Aβ) treatment led to an augmented number of ER-mitochondrial contacts in hippocampal neurons [84].

### APOE in mitochondria-associated ER membranes

Vance [17] showed in 1990 that apolipoproteins such as apolipoprotein B (APOB) and apolipoprotein C (APOC), but particularly APOE, were enriched in MAM fractions isolated from rat liver. APOE was also detected in the mitochondrial fraction, but remarkably 15-fold higher in MAM fractions indicating a MAM resident function of APOE [17]. Analyses of the MAM proteome by subcellular isolation of MAM fractions and subsequent liquid chromatography–mass spectrometry (LC–MS) studies in mouse liver, brain, muscle, testes, Huh7 cells, and human testes also indicated the presence of APOE in MAM fractions (Table 1) together with hundreds of other proteins [30, 34–36, 85]. With the exception of Vance [17], previous work has focused on untargeted proteomics to investigate the general composition of MAMs. It is still poorly understood how APOE localizes to MAMs, whether it is permanently present or only during certain conditions, and what physiological function APOE has there. The possible functions of APOE described in the literature and the effects of APOE isoforms on MAMs are shown in Table 2 and Fig. 2b. One possible explanation for how APOE enters the MAM is the physical interaction with the MAM resident proteins GRP75 and VDAC1 [51, 52, 86]. Apart from the protein–protein interaction, the level of different GRP75 isoforms and their phosphorylation have been shown to be regulated in part depending on the APOE isoform. For example, GRP75 isoform c was strongly upregulated in the presence of APOE4, whereas the expression of isoform d was increased with APOE3 [66]. Other authors did not distinguish between GRP75 isoforms and showed downregulation of GRP75 protein expression in APOE4 Neuro2a cells [57] and no effect of APOE on GRP75 protein levels in cultured brain cortical neurons and astrocytes [58]. A further link between APOE and GRP75 became apparent when an APOE4 fragment (amino acids 1–271) was examined, as the fragment caused ER stress and increased GRP75 expression, presumably leading to increased MAM formation and mitochondrial calcium overload [42]. Whether APOE3 fragments have the same effect was not investigated.

Recombinant APOE, which is bound to a specific LDL form, was found to colocalize with VDAC1 after lysosomal degradation of LDL, and leads to increased opening of the mitochondrial permeability transition pore (mPTP) and mitochondrial swelling [52]. This finding could indicate a role of APOE in the regulation of mitochondrial function via the interaction with VDAC1. Multiple proteins have been identified that bind to VDAC1, and some of them regulate VDAC1 function. For example, VDAC1 closure is regulated through the binding of tubulin [87]. It may be conceivable that the binding of APOE to VDAC1 affects the function of the VDAC1–GRP75–IP3R1 complex and, as a result, the shuttling of calcium through MAMs. Increased calcium flux from the ER to the mitochondrion was observed in APOE4 Neuro2a cells compared with APOE3 cells [57], but whether this is the result of a physical interaction of APOE with the VDAC1–GRP75–IP3R1 complex remains to be investigated systematically.

Apart from the physical interaction of APOE with MAM resident proteins, there is evidence for a role of APOE in one of the major functions of mitochondria–ER contacts, the lipid metabolism. Increased conversion of phosphatidylserine (PS) to phosphatidylethanolamine (PE) was observed in human fibroblasts and mouse hippocampus cells treated with APOE4-containing astrocyte-conditioned media compared to APOE3 [67]. However, the protein expression level of the respective enzyme catalysing the conversion from PS to PE was not altered in response to the APOE isoform. The APOE isoform-dependent effect on phospholipid metabolism was not visible in MFN2 knockout cells (decreased ER mitochondrial communication). The authors interpreted this as an indication that the APOE4-dependent effect on phospholipid metabolism is due to increased ER-mitochondrial communication in APOE4-treated cells (defined as MAM activity) [67]. When comparing the hepatic MAM proteome of caveolin 1 (CAV1; main component for caveolae formation) knockout mice and wild-type mice, it was found that APOE protein levels were decreased in the MAM fraction of CAV1 knockout mice. At the same time, increased cholesterol levels were observed in MAM fractions of CAV1 knockout mice [30]. The authors hypothesize that the reduced presence of APOE in MAMs of CAV1 knockout mice disturbs the secretion of VLDL, leading to increased cholesterol levels. There is further evidence of a relationship among cholesterol metabolism, APOE, and the protein composition of MAMs, as shown by an examination of a complex of rough ER wrapped around mitochondria containing MAMs as a subdomain [37]. Transcriptomics and proteomics of these ER-mitochondria complexes in mouse liver revealed an enrichment of proteins involved in VLDL biogenesis, including APOE [37]. These examples demonstrate the possible participation of APOE in phospholipid and cholesterol metabolism in MAMs.

The influence of the APOE isoform on MFN1 and MFN2 expression, which are important representatives of MAM tethering proteins, has been investigated in several studies. In APOE-transfected Neuro2a cells, primary astrocytes from APOE TR mice, and the hippocampus of APOE TR mice, higher MFN1 protein levels were observed in APOE4 [29, 57, 61]. This finding could indicate a possible increased connection between the ER and mitochondria in APOE4 [57]. However, in the human postmortem brain, higher protein levels were found in *APOEε3* carriers [62], and in Huh7 cells and APOE TR mouse liver, no differences were found at the mRNA level [53].

Overall, there is some evidence of the participation of APOE in various pathways in MAMs through physical appearance [17], interactions with MAM proteins [52, 86], and influences on MAM functions [30, 42, 67]. These studies provide the first clues about the possible role of APOE in mitochondria–ER contacts, but more systematic research is needed. An overview of the described possible functions of APOE in mitochondria and MAMs is given in Fig. 2b.

## Protein–protein interactions

Protein–protein interactions play a crucial role in normal cell function and have been gained scientific interest for decades. The analysis of the human interactome network, for example, has identified up to 100,000 protein–protein interactions [88]. A prominent example of an APOE protein–protein interaction of central importance to its role in lipid metabolism, is the binding to receptors of the LDLR family [89]. A prerequisite for the interaction, however, is the exposure of the specific receptor-binding site in the N-terminus of APOE that is only exposed upon lipid binding [90]. The APOE protein consists of a N- and a C-terminal domain that interact through numerous amino acid residues. There have been a few APOE isoform-dependent differences reported concerning the domain dissociation, folding intermediate formation, and the size of preferentially bound lipid particles [22, 23, 91]. Therefore, it is conceivable that there also exist differences in protein–protein interactions, owing to the differences in binding site exposure, between the APOE isoforms.

### APOE protein–protein interactions beyond lipid metabolism

**Fig. 3:**
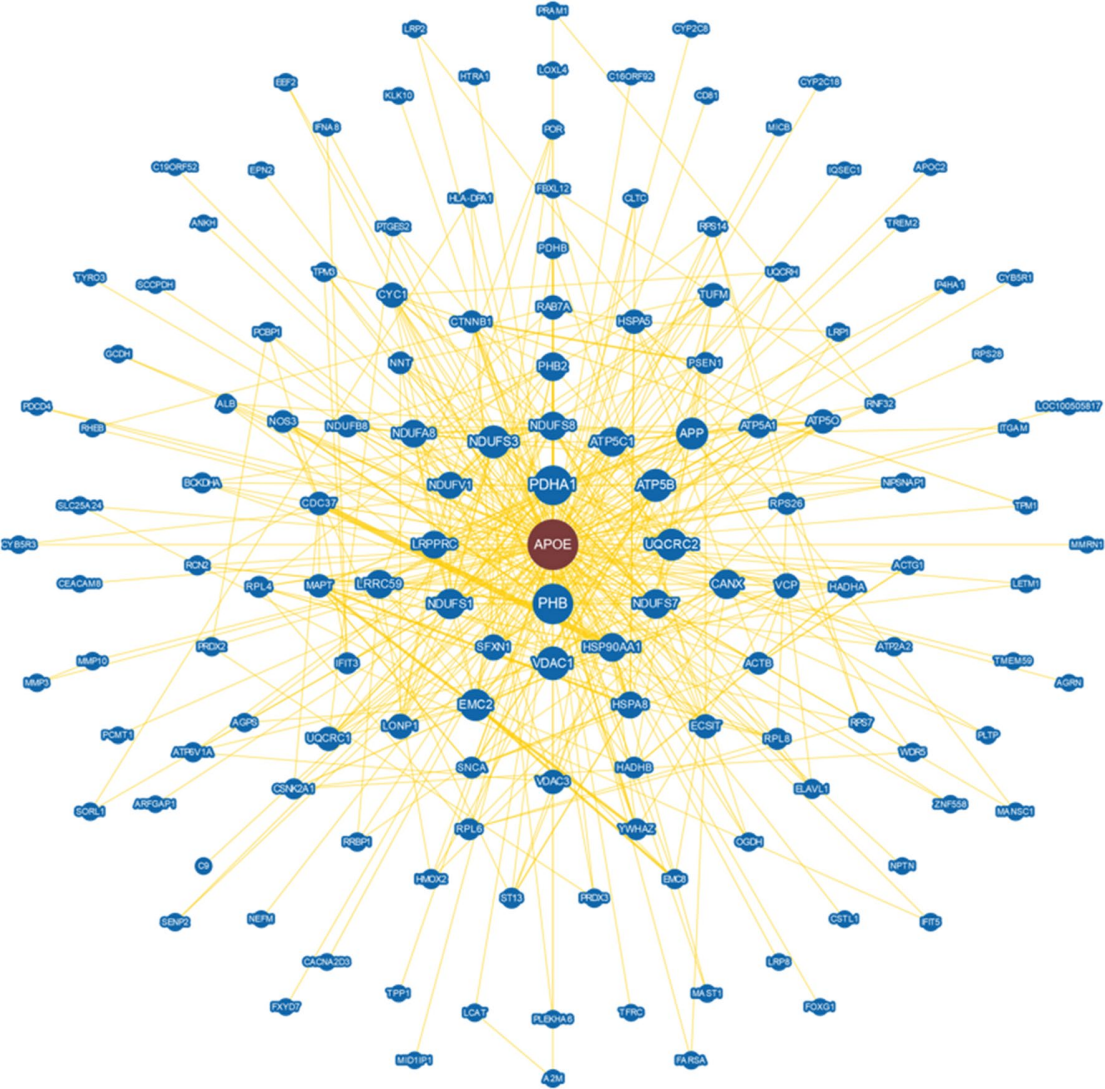
Potential APOE protein–protein interactions in human cells and tissue. An in silico analysis performed with BioGRID 4.4 software revealed that there is evidence for 145 potential protein binding partners whose physical interaction with APOE has been demonstrated in at least one study in each case. The larger the blue circle of the corresponding protein is, the stronger the connectivity with APOE, and thicker binding lines represent stronger evidence supporting the association. Image downloaded from https://thebiogrid.org/

By studying novel protein–protein interactions of APOE, new metabolic pathways involving APOE may be discovered, and potentially novel functions of APOE, apart from its major role in lipid transport and clearance, may be identified. According to PubMed and the BioGRID 4.4 database (Database of Protein, Genetic and Chemical Interactions, https://thebiogrid.org/), approximately 150 proteins interacting with APOE have been identified in human cells and tissues to date. The protein–protein interaction network of an in silico analysis (BioGRID) of the APOE interaction partners discovered thus far is shown in Fig. 3 and illustrates the large number of potential binding partners that may indicate metabolic pathways with previously unknown APOE involvement. Apart from the interactions of APOE with LDLR and other proteins involved in cholesterol metabolism, several other APOE protein–protein interactions have recently been identified. However, the majority of the large number of potential APOE interaction partners that were detected by untargeted so-called “high-throughput” interactome studies, which identify a wide variety of different interactions, have not yet been further investigated for their biological relevance in functional studies or independently confirmed. For example, in 2015, Huttlin et al. [51] published an interactome study in which they identified over 20,000 protein–protein interactions in HEK293T cells by affinity purification and MS, of which 86% of these interactions were previously unknown, including the interactions of APOE with the major histocompatibility complex class II DPα1, matrix metallopeptidase 3, multimerin 1, and VDAC1 [51]. These four proteins were also repeatedly identified as APOE interaction partners in two subsequent interactome studies by Huttlin et al. [92, 93]. Considering only the mitochondrial interactome, APOE was found to be associated with branched-chain alpha-keto acid dehydrogenase subunit α (BCKDHA), subunits of F_1_-ATP synthase and other respiratory chain complexes (e.g., cytochrome b–c1 complex subunit 1 and 2 (UQCRC1/2), and subunits of NADH dehydrogenase) as well as nonmitochondrial proteins such as cytoplasmic actin, and calnexin, which is located in the ER membrane, among others [94].

### Interactions of APOE with nonmitochondrial proteins

Functional studies have already been performed on some APOE protein–protein interactions, partly investigating isoform-dependent differences or examining the physiological significance. These “low-throughput” functional studies are listed in Table 3. Since a markedly large number of mitochondrial proteins were found in the interactome studies and some of them were characterized in more detail, Table 3 is divided into interactions of APOE with nonmitochondrial and mitochondrial proteins. APOE protein–protein interactions with nonmitochondrial proteins include proteins associated with AD pathology, such as the amyloid-β precursor protein (APP) [95, 96]. A network of AD-relevant proteins that interact with each other has been identified by Soler-López et al. [97], which may provide further evidence to elucidate the underlying mechanisms of AD. In addition to AD pathology, APOE has also been shown to bind to synuclein α (SNCA), which accumulates in Parkinson’s disease [98, 99]. However, several other proteins that are not directly related to diseases have been further investigated regarding their interaction with APOE. These include haptoglobin (HPT), which is a haemoglobin binding and transport protein in the plasma and has been shown to bind to APOE via a specific binding site in the APOE amino acid sequence (amino acids 131–150) [100–102].

**Table 3:** Overview of “low-throughput” studies in which targeted APOE protein–protein interactions were investigated and characterized for their biological function

Interacting proteins	Methods	Tissue/cells	Biological relevance	References
**Nonmitochondrial**				
A2M	Co-IP WB	Human plasma	Interaction E2 = E3 > E4 Interaction affected by lipoproteins	[103]
APP	Co-IP WB Affinity chromatography WB	COS-1 cells Human plasma	APP is the precursor of Aβ, which plays an important role in AD pathology due to its neurotoxic properties and accumulation in plaques	[95] [96]
ABCA1	Co-IP WB	Human skin fibroblasts, human plasma	Interaction E2 = E3 = E4 APOE might act as a protector for ABCA1 against proteases	[104] [105]
HPT	HPT-coupled beads, loaded with human plasma, pulldown, WB	Human plasma	Interaction of APOE and HPT via a specific amino acid sequence in APOE (amino acids 131–150) HPT potentially enhances the formation of the APOE–Aβ complex, may be related to AD pathology Inhibition of LCAT activity	[100,101, 102]
NOS1	Co-IP WB Coimmunostaining	HEK293 cells Human hippocampus	Interaction E2 = E3 = E4 Interaction potentially leads to *S*-nitrosylation of APOE2 and APOE3, but not APOE4	[106]
CDC37 ECSIT PDCD4 ST13	Co-IP WB	SH-SY5Y cells COS-7 cells	All investigated proteins are related to AD pathology Identification of these protein–protein interactions provides a contribution to the elucidation of possible AD-relevant mechanisms	[97]
SNCA	Co-IP WB Co-IP ELISA	Human plasma Human CSF	SNCA accumulation is a maker for Parkinson’s disease pathology SNCA interacts with APOE and other apolipoproteins Increased APOE level in CSF from Parkinson’s disease patients	[98] [99]
**Mitochondrial**				
ATP5A/B	Affinity chromatography WB	Human liver	Potential function in mitochondrial metabolism	[107]
UQCRC1/2 CYC1	Co-IP WB	Neuro2a cells	Interaction E4 fragment > E4 full-length APOE3 was not investigated APOE4 fragment alters mitochondrial activity	[33]
GRP75	Co-IP WB	Astrocytes from APOE TR mice Human hippocampus	Interaction only in E4 cells, not in E3 and E2 Interaction in hippocampus E3 control < E3 AD < E4 AD Different GRP75 isoforms are regulated differently depending on the APOE isoform	[86] [66]
VDAC1	Co-IP WB Coimmunostaining	H9c2 cells	Interaction E3 = E4 Interaction of APOE (derived from the lysosomal degradation of L5-LDL) with VDAC1 induced mitochondrial swelling (higher in APOE4)	[52]

*Co-IP* co-immunoprecipitation, *WB* Western blot, *ELISA* enzyme-linked immunosorbent assay, *A2M* α2-macroglobulin, *APP* amyloid precursor protein, *ABCA1* ATP-binding cassette transporter, *HPT* haptoglobin, *NOS1* nitric oxide synthase 1, *CDC37* cell division cycle 37 *HSP90* cochaperone, *ECSIT* evolutionarily conserved signalling intermediate in the toll pathway, *PDCD4* programmed cell death 4, *ST13* suppression of tumorigenicity 13 protein, *SNCA* synuclein α, *ATP5A/B* ATP synthase subunit α/β, *UQCRC1*/2 cytochrome b–c1 complex subunit 1/2, *CYC1* cytochrome C1, *GRP75* glucose-regulated protein 75, *VDAC1* voltage-dependent anion-selective channel 1

### Interactions of APOE with mitochondrial proteins

Interestingly, among the APOE protein–protein interactions identified thus far are many mitochondrial proteins (Table 3), although APOE is a secretory protein. Investigation of the mitochondrial interactome of human pluripotent embryonal carcinoma stem cells revealed 54 proteins associated with APOE [94]. Some of these interactions have been described before, while others are completely unknown. The interactome study showed the protein–protein interaction of APOE with eight subunits of complex I and with four subunits each of complex III and complex V/F_1_-ATP synthase of the mitochondrial electron transport chain [94]. The association of APOE with the α- and β-subunits of F_1_-ATP synthase, UQCRC1/2, and cytochrome C1 (CYC1) has been partly described previously in human liver tissue and APOE-transfected Neuro2a cells [33, 107]. In 1988, APOE was shown to be associated with high affinity for a protein complex in the human liver [107]. The researchers identified the α- and β-subunits of F_1_-ATP synthase as components of this complex and demonstrated for the first time the interaction of APOE with mitochondrial proteins [107]. Twenty years later, these proteins were again discovered among APOE-associated proteins in APOE4-transfected Neuro2a cells [33]. A C-terminally truncated APOE4 fragment interacted more strongly with the mitochondrial proteins studied than did full-length APOE4. The reason for this could be a more altered spatial structure compared to full-length APOE. Since APOE3 was not studied, no conclusion can be made about potential isoform-dependent differences in terms of the interaction with mitochondrial proteins [33]. The impact of the APOE isoform on mitochondrial metabolism has already been investigated in several studies, especially in the brain and neuronal cells (Table 2), but whether the interaction of APOE with mitochondrial proteins of the electron transport chain is reflected in mitochondrial function, e.g., oxidative phosphorylation or ATP levels, or whether it is causal for disease-associated mitochondrial dysfunction still needs to be explored.

Another interactome study in HEK293T cells emphasized the association of APOE with the mitochondrial channel protein VDAC1, which is localized in the OMM [51]. Based on this information, Chen et al. [52] investigated the effect of the VDAC1–APOE interaction on mitochondrial function in rat cardiomyocytes (H9c2 cells). They treated H9c2 cells with recombinant APOE3 or APOE4 and confirmed the interaction of APOE and VDAC1 by co-immunoprecipitation (co-IP) and immunostaining experiments. They concluded that APOE, derived from the lysosomal degradation of a highly electronegative subtype of LDL (L5), influences the opening and closing of the mPTP, leading to mitochondrial swelling [52]. The interaction with VDAC1 was comparable in APOE3- and APOE4-treated cells, but mitochondrial swelling was greater in APOE4-treated cells.

A large number of proteins have already been identified to interact with VDAC1, including GRP75, which has also been shown to interact with APOE in astrocytes from APOE TR mice and in the human hippocampus [86]. The functional relevance of the GRP75–APOE interaction has not been further examined, but the interaction was observed only in APOE4 astrocytes (not in APOE3 and APOE2) and was different in the human hippocampus depending on the AD or non-AD status and APOE isoforms, with the lowest interaction observed in non-AD *APOEε3* carriers, followed by *APOEε3* AD, and the highest in *APOEε4* AD [86]. Rather than considering the interactions of APOE with GRP75 and VDAC1 separately and assuming that they are dimer protein–protein interactions, these results may also indicate a larger protein complex involving APOE. VDAC1 and GRP75, together with IP3R1, are involved in calcium transport in MAMs (Fig. 2b), which may indicate a role for APOE in this protein complex and thus in mitochondrial calcium homeostasis.

We were able to demonstrate the interaction of APOE with VDAC1 in the liver of APOE TR mice, and its upregulation in response to dietary restriction in APOE3 and APOE4 mice. Another interactor protein identified in this study was BCKDHA, a subunit of an enzyme complex involved in the catabolism of branched-chain amino acids (BCAA). Remarkably, the enzyme activity of this complex was increased in obese APOE4 mice compared to APOE3 mice, but without an effect on plasma BCAA levels in (manuscript in preparation, Rueter et al. 2022).

By identifying and characterizing APOE protein–protein interactions, it becomes clear what multiple potential non-canonical functions of APOE may exist. Many mitochondrial proteins that potentially bind to APOE have been identified in interactome studies, but only a few have been investigated for their biological relevance. Thus, many unanswered questions remain, such as whether protein–protein interactions are permanent or transient and whether they are modifiable by physiological factors, including diet.

## Conclusion

In conclusion, novel findings considering its subcellular localization and its unrecognized interaction with other proteins warrants a revision of the traditional view on APOE solely as mediator of lipoprotein-receptor binding. Regarding the large body of evidence existing on the mitochondrial localization, APOE isoform-dependent regulation of mitochondrial functions, and the interaction with mitochondrial proteins, a participation of APOE in the functional network of mitochondria is strongly suggested. However, it remains to be elucidated, by which transfer mechanisms APOE enters the inner mitochondrial matrix and whether this occurs during the process of mitochondria-associated proteostasis. The latter implies that APOE is either proteolytically cleaved or recognized as misfolded, prior to its mitochondrial incorporation. An isoform-dependent mitochondrial accumulation of proteolytic fragments and misfolded intermediates must be considered and may explain the adverse effects on mitochondrial function attributed to APOE4.

The question of whether the association of APOE isoforms with mitochondrial and MAM function has an impact on human health requires further research. Mitochondrial dysfunction in the brain of *APOEε4* carriers is discussed as contributing to the development of AD, but it remains unclear whether APOE4 also leads to impaired mitochondrial function in other organs, which would be fatal since mitochondria are essential cell organelles for central processes such as energy metabolism. Because other proteins of lipoprotein metabolism besides APOE have been localized in the MAMs, it is reasonable to assume that APOE plays a role in VLDL synthesis in the hepatic MAMs. Since the lipoprotein binding preference differs between the APOE isoforms and APOE4 has a higher preference for VLDL than APOE3, differences in lipid metabolism in the MAMs may result.

The functional importance of mitochondria–ER contacts has been increasingly recognized and there is growing evidence for a central involvement of APOE. MAM assembly and protein distribution is affected by the cellular energy state and nutritional factors such as high glucose levels, while the *APOE* genotype has been observed to modulate the responsiveness to dietary energy and micronutrients. Therefore, focussing on APOE isoform-dependent MAM dynamics in response to the availability of cellular energy substrates or dietary micronutrients can be a valuable approach to future research. However, when selecting the target tissue to study the function of APOE in MAMs and mitochondria, it should be noted that the APOE isoproteins might have a different effect in the brain than in the rest of the body, including the liver. In this regard it is also conceivable that the composition and functional dynamics of MAMs differ depending on the type of tissue analysed. Therefore, the direct comparison of APOE isoform-dependent modifications of mitochondrial and MAM function in the brain versus the liver is inevitable.

## Data Availability

Not applicable.

## Abbreviations

A2M: α2-Macroglobulin
ABCA1: ATP-binding cassette transporter
AD: Alzheimer’s disease
APOB: Apolipoprotein B
APOC: Apolipoprotein C
APOE: Apolipoprotein E
APP: Amyloid-β precursor protein
ATP5A/B: ATP synthase subunit α/β
BCAA: Branched-chain amino acid
BCKDHA: Branched-chain alpha-keto acid dehydrogenase subunit α
CAV1: Caveolin 1
CDC37: Cell division cycle 37, HSP90 cochaperone
CSF: Cerebrospinal fluid
Co-IP: Co-immunoprecipitation
CYC1: Cytochrome C1
DRP1: Dynamin-related protein 1
ECSIT: Evolutionarily conserved signalling intermediate in Toll pathways
ELISA: Enzyme-linked immunosorbent assay
ER: Endoplasmic reticulum
FIS1: Mitochondrial fission 1 protein
GRP75: Glucose-regulated protein 75
HPT: Haptoglobin
IMM: Inner mitochondrial membrane
IP3R1: Inositol 1,4,5-trisphosphate receptor 1
LC–MS: Liquid chromatography–mass spectrometry
LDL: Low-density lipoprotein
LDLR: Low-density lipoprotein receptor
MAMs: Mitochondria-associated ER membranes
MFN1/2: Mitofusin 1/2
MLS: Mitochondrial localization sequence
mPTP: Mitochondrial permeability transition pore
MS: Mass spectrometry
NLS: Nuclear localization sequence
NOS1: Nitric oxide synthase 1
OMM: Outer mitochondrial membrane
OPA1: Dynamin-like 120 kDa protein
OXPHOS: Oxidative phosphorylation
PC: Phosphatidylcholine
PDCD4: Programmed cell death 4
PE: Phosphatidylethanolamine
PEMT: Phosphatidylethanolamine methyltransferase
PGC1α: Peroxisome proliferator-activated receptor gamma coactivator 1-α
PS: Phosphatidylserine
PSD: Phosphatidylserine decarboxylase
PSS1/2: Phosphatidylserine synthase 1/2
SIRT3: Sirtuin 3
SNCA: Synuclein α
ST13: Suppression of tumorigenicity 13 protein
TCA: Tricarboxylic acid cycle
TIM: Translocase of the inner mitochondrial membrane
TOM: Translocase of the outer mitochondrial membrane
TR: Targeted replacement
UQCRC1/2: Cytochrome b–c1 complex subunit 1/2
VDAC1: Voltage-dependent anion-selective channel 1
VLDL: Very low-density lipoprotein
WB: Western blot
